# Using Perls Staining to Trace the Iron Uptake Pathway in Leaves of a Prunus Rootstock Treated with Iron Foliar Fertilizers

**DOI:** 10.3389/fpls.2016.00893

**Published:** 2016-06-27

**Authors:** Juan J. Rios, Sandra Carrasco-Gil, Anunciación Abadía, Javier Abadía

**Affiliations:** Department of Plant Nutrition, Aula Dei Experimental Station, Consejo Superior de Investigaciones CientíficasZaragoza, Spain

**Keywords:** *Prunus dulcis × P*. *persica*, Fe plant nutrition, foliar Fe fertilization, leaf Fe localisation, Perls blue staining

## Abstract

The aim of this study was to trace the Fe uptake pathway in leaves of Prunus rootstock (GF 677; *Prunus dulcis* × *Prunus persica*) plants treated with foliar Fe compounds using the Perls blue method, which detects labile Fe pools. Young expanded leaves of Fe-deficient plants grown in nutrient solution were treated with Fe-compounds using a brush. Iron compounds used were the ferrous salt FeSO_4_, the ferric salts Fe_2_(SO_4_)_3_ and FeCl_3_, and the chelate Fe(III)-EDTA, all of them at concentrations of 9 mM Fe. Leaf Fe concentration increases were measured at 30, 60, 90 min, and 24 h, and 70 μm-thick leaf transversal sections were obtained with a vibrating microtome and stained with Perls blue. *In vitro* results show that the Perls blue method is a good tool to trace the Fe uptake pathway in leaves when using Fe salts, but is not sensitive enough when using synthetic Fe(III)-chelates such as Fe(III)-EDTA and Fe(III)-IDHA. Foliar Fe fertilization increased leaf Fe concentrations with all Fe compounds used, with inorganic Fe salts causing larger leaf Fe concentration increases than Fe(III)-EDTA. Results show that Perls blue stain appeared within 30 min in the stomatal areas, indicating that Fe applied as inorganic salts was taken up rapidly *via* stomata. In the case of using FeSO_4_ a progression of the stain was seen with time toward vascular areas in the leaf blade and the central vein, whereas in the case of Fe(III) salts the stain mainly remained in the stomatal areas. Perls stain was never observed in the mesophyll areas, possibly due to the low concentration of labile Fe pools.

## Introduction

Iron deficiency is a limiting factor for food production in many areas of the world, affecting agricultural produce quality and yield in horticultural and fruit tree crops (Abadía et al., 2011; Briat et al., 2015). Species affected include grapevine, citrus, pear and peach trees and others. In Fe-deficient plants leaves become yellow because the synthesis and assembly of thylakoid components are impaired (Terry and Abadía, 1986).

Different agricultural management strategies are used to control Fe chlorosis in tree crops, with the most common one being the soil application of soluble Fe(III)-chelates (El-Jendoubi et al., 2011). Other current practices include the injection of Fe compounds in liquid or solid forms into tree branches (Larbi et al., 2003), as well as the application of fertilizers to the plant foliage, the so-called foliar fertilization (Fernández et al., 2013). Foliar fertilization is cheaper than soil fertilization, although the effectiveness of the technique may, in some cases, be insufficient to correct Fe deficiency (El-Jendoubi et al., 2014). Both inorganic- and organic-based Fe compounds are currently used in foliar fertilization (Abadía et al., 2011; Fernández et al., 2013). Until now, foliar Fe fertilization studies have been focused on testing which Fe formulations have a better efficiency (e.g., Fe oxidation states, ions vs. chelates, pH, surfactants and adjuvants, etc.; Rombolà et al., 2000; Abadía et al., 2002; Álvarez-Fernández et al., 2004; Fernández and Ebert, 2005; Schönherr et al., 2005; Fernández et al., 2006; Fernández and Eichert, 2009; Borowski, 2013), understanding the Fe uptake pathway in the leaf and investigating whether environmental conditions could affect Fe uptake from fertilizers (Eichert and Burkhardt, 2001; Schönherr, 2001; Schönherr and Luber, 2001; Schlegel and Schönherr, 2002; Schönherr and Schreiber, 2004; Fernández and Ebert, 2005; Schönherr et al., 2005).

Many foliar fertilization studies have compared Fe chemical forms. Early studies provided conflicting data, since some of them indicated that FeSO_4_ had better entrance rates than Fe(III)-EDDHA (Kannan, 1969), whereas others observed that chelates were faster than inorganic Fe regarding penetration and translocation (Basiouny and Biggs, 1976). Recent foliar fertilization studies with Fe-deficient pear trees, including surface-active agents, indicated that FeSO_4_ had a similar re-greening effect to that of Fe(III)-DTPA (Álvarez-Fernández et al., 2004). In peach trees treated with different Fe-compounds, the best regreening results were with FeSO_4_, followed by those with Fe(III)-citrate and Fe(III)-EDTA (Álvarez-Fernández et al., 2004).

Environmental conditions, including temperature, relative humidity (RH) and light, could affect leaf Fe uptake (Ramsey et al., 2005). Temperature could influence chemical reactions and physical proprieties of plants from the cellular to the whole plant level (Gruda, 2005) and moderately high temperatures could stimulate photosynthesis, transpiration and penetration rates, although the permeability of isolated leaf cuticles to salts was shown to be unaffected by temperature (Schönherr and Luber, 2001; Schönherr, 2002). Also, light affects many leaf physiological processes such as stomatal opening, photosynthesis and xylem flux. Open stomata could increase foliar uptake (Outlaw, 2003) and this has been described as a major factor for foliar penetration through the abaxial leaf side (Greenne and Bukovac, 1971; Schlegel et al., 2006). Another key factor is RH, which could influence many processes, including the hydration of leaf cuticles, which decrease with decreasing RH (Chamel et al., 1991, 1992; Schönherr, 2000), as well as the applied fertilizer drying time and/or deliquescence on the leaf surface (the point of deliquescence, POD, is defined as the RH value when the compound becomes a solute; Fernández et al., 2008; Fernández and Eichert, 2009). Many studies on foliar fertilization were carried out under 100% RH to simplify the system (Schönherr and Luber, 2001; Schönherr and Schreiber, 2004; Eichert and Goldbach, 2008), although such RH values are rarely found -even at night- under field conditions in agricultural areas affected by Fe deficiency.

Leaves have a protecting cuticle that limits the transport of water and ions, not only at its outer surface, composed of cutin and waxes (Schönherr and Riederer, 1986; Schönherr and Schreiber, 2004), but also possibly due to its internal structure and composition (Fernández et al., 2016). The cuticle minimizes passive water loss and avoids leaching of apoplastic solutes. It has been suggested that hydrophilic compounds (including Fe) may be taken up by leaves through cuticle cracks, stomata, leaf hairs and perhaps through specialized epidermal cells (Fernández and Brown, 2013), and it is known that physical damage of the leaf surface could aid penetration (Jordan and Brodribb, 2007; Munné-Bosch, 2007). It has been also hypothesized that hydrophilic solutes could penetrate cuticles by a pathway different than that used by hydrophobic solutes, following the so-called “polar pores” or “aqueous pores”, generated by the adsorption of water to polar moieties located in the cuticle (Schönherr, 2000; Schönherr and Schreiber, 2004); however, these pores have not been visualized so far (Koch and Ensikat, 2008).

Uptake of pure water solutions through stomata was considered unlikely by Schönherr and Bukovac (1972), but further studies indicated that condensation water on the pore walls may facilitate stomatal uptake (Eiden et al., 1994; Burkhardt et al., 1999), and that uptake via stomata could occur not only for large anions such as uranine but also for small cations such as Fe(III) (Eichert and Burkhardt, 2001). Image studies were carried out using different techniques, including fluorescent dyes. Whereas Strugger (1939) indicated that the dye was visible inside stomata, and Dybing and Currier (1961) showed that an anionic fluorescent dye entered leaves *via* stomata, Schönherr and Bukovac (1978) and Schönherr (2006) indicated that cuticular ledges were preferential points, perhaps having a higher permeability, for penetration of solutes accumulated in vicinity of guard cells. The role of stomata in foliar uptake was also supported by the enhanced uptake of fluorescein in leaves when stomata were open (Eichert et al., 1998). Also, Eichert and Burkhardt (2001) and Eichert et al. (2008) have suggested the occurrence of active and inactive stomata in terms of cuticle wettability around guard cells and solute uptake.

The Perls Prussian blue stain, widely used to detect labile Fe in biological tissues by forming a precipitate with a formula Fe_4_[Fe(CN)_6_]_3_ x H_2_O [the corresponding IUPAC name is Iron(II,III) hexacyanoferrate(II,III)], has been applied only recently to plant research, due to poor penetration and sensitivity in hydrophobic tissues. Whereas the Perls method has been reported to stain Fe at concentrations of 35 μM from both Fe(II) and Fe(III) (Roschzttardtz et al., 2009), a new protocol, with an additional step including diaminobenzidine, was designed to enhance sensitivity (Roschzttardtz et al., 2009, 2010). This was later applied to leaves treated with Fe foliar fertilizers (El-Jendoubi et al., 2014).

In summary, in spite of the many studies carried out on foliar fertilization, the knowledge on the Fe uptake pathways in leaves is still poorly known. The aim of this study was to investigate how Fe applied to the leaf surface in different chemical forms enters the leaves of a *Prunus* species, using Perls staining, a technique capable to detect labile Fe pools. The rootstock GF677 was used as a *Prunus* model because of the commercial agricultural interest and the easiness in getting adequate plant material.

## Materials and Methods

### Plant Growth Conditions and Sampling

Micropropagated, clonal GF 677 rootstock plants [*Prunus dulcis* (Mill.) D.A. Webb × *Prunus persica* (L.) Batsch] were acquired from Agromillora Catalana S.A. (Subirats, Barcelona, Spain). Plants were grown for 2 weeks in 300 mL pots on a peat substrate. Plants were transferred for 2 days to 15 L plastic boxes (31 plants per box) filled with Hoagland’s nutrient solution diluted 10-fold. Then, plants were grown in continuously aerated, half-strength Hoagland nutrient solution pH 5.5, containing (in mM) 2.5 Ca(NO_3_)_2_, 2.5 KNO_3_, 1 MgSO_4_, 1 KH_2_PO_4_, and (in μM) 46.2 H_3_BO_3_, 9.2 MnCl_2_, 0.38 CuSO_4_, 2.4 ZnSO_4_, 0.12 Na_2_MoO_4_, and 90 μM Fe(III)-EDTA. Plants were grown in a growth chamber (Fitoclima 10000 EHHF, Aralab, Albarraque, Portugal) with a photoperiod of 16 h light (with a PPFD of 350 μmol photon m^-2^ s^-1^ PAR at the leaf level) at 23°C/8 h of darkness at 20°C, and constant 70% RH. Nutrient solutions were renewed every week. After 2 weeks of growth, when roots were approximately 10 cm long (with 5–6 leaves), plants were transferred to 2 L pots (four plants per pot) filled with half-strength Hoagland nutrient solution, pH 5.5, containing 0 μM [-Fe] or 90 μM Fe(III)-EDTA [+Fe]. Subsequently, nutrient solutions were renewed every week. Iron deficiency symptoms (leaf chlorosis) appeared progressively in young leaves.

**FIGURE 1:**
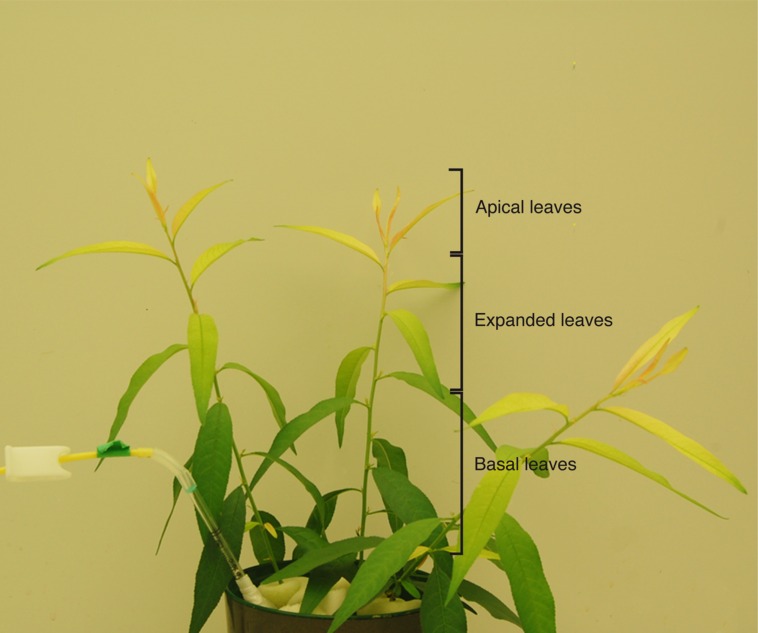
***Prunus dulcis × P. persica* plants grown in nutrient solution under Fe deficiency for 15 days.** Iron-deficiency symptoms include leaf chlorosis. Expanded leaves were those selected to be treated with the Fe foliar fertilizers.

Treatments were applied to leaves of plants grown under Fe deficiency conditions for approximately 2 weeks, when plants had approximately 11 leaves (**Figure 1**). Foliar fertilization was carried out in expanded Fe-deficient leaves with a SPAD reading of approximately 10–20 (green, Fe-sufficient leaves had at this stage SPAD values of approximately 35). Plants were treated with foliar fertilizers approximately 2 h after the onset of the light period in the growth chamber. Many preliminary experiments were carried out to explore the possibility of detecting Fe with Perls, and it was observed that a dark-pretreatment led to the best results. Therefore, the standard protocol included covering plants with a black plastic bag overnight, with the bag being removed only 30 min before foliar application. In the case of FeSO_4_, the treatment was also applied without this dark pre-treatment, i.e., in plants illuminated by the growth chamber light for 2 h. Four different fertilizer solutions containing 9 mM Fe were used. The experiment used a completely randomized design with three replications. The solutions contained: (i) the Fe(II) salt FeSO_4_; (iii) the Fe(III) salt Fe_2_(SO_4_)_3_; (iv) the Fe(III) salt FeCl_3_, and (ii) the chelate Fe(III)-EDTA; all solutions contained 0.2% of a non-ionic, organo-silicon surfactant (Break-Thru S 233, Evonik Industries AG, Essen, Germany). The products used were FeSO_4_.7H_2_O (CAS Number 7782-63-0; Sigma–Aldrich), Fe_2_(SO_4_)_3_.xH_2_O (CAS Number 15244-10-7), and FeCl_3_.6H_2_O (CAS Number 10025-77-1).

The pH values after adding the surfactant were 3.9, 3.3, 3.2, and 4.1 in the cases of FeSO_4_, Fe_2_(SO_4_)_3_, FeCl_3_, and Fe(III)-EDTA, respectively, and no further pH adjustments were made to comply with the normal grower’s practices. A similar pH value, 4.0, has been used for of FeSO_4_ in previous studies (Fernández et al., 2008; El-Jendoubi et al., 2014). The fertilizer was applied only to the abaxial side of five fully expanded Fe-deficient leaves in each plant using a paintbrush (in this plant species stomata are only present in the abaxial side). The procedure was repeated three times, with the full fertilization process lasting approximately five min. The total volume of foliar fertilizer (the sum of the three applications) was approximately 700 μL per plant. To test the role of stomata, the nutrient solution of one pot was supplemented with 100 μM ABA (from stock dissolved in methanol) 24 h before foliar fertilization. Leaf samples were taken for mineral and microscopic analysis at four different times after the first foliar fertilizer application: 30, 60, and 90 min and 24 h.

### Analysis of Micronutrient Concentrations

Leaves (fertilized and untreated controls) were washed thoroughly twice with ultrapure water Type I. Leaves were dried in an oven at 60°C, ground in a ZrO_2_ ball mill (MM301, Retsch, Haan, Germany) and stored at room temperature until analysis. Plant samples (200 mg DW of tissue) were digested using a microwave system (Milestone Ethos Plus, Bergamo, Italy) with 6.4 mL HNO_3_ (26%, TraceSelect Ultra, Sigma–Aldrich) and 1.6 mL H_2_O_2_ (30%). The microwave digestion program was 5 min at 100°C, 10 min at 170°C, and 35 min at 180°C. The digest was filtered through a 0.45 μm PTFE filter, diluted to 10 mL in water Type I, and metals (Fe, Mn, Cu, and Zn) determined by flame atomic absorption spectrometry (FAAS) using a Solaar 969 apparatus (Unicam Ltd, Cambridge, UK). Three replications per treatment and batch were analyzed. Total micronutrient contents in leaves were obtained from the leaf concentrations and DW values. Iron concentration data were analyzed using two-way ANOVA and means compared (Fisher’s LSD test at *p* < 0.05) using Genstat.

### Leaf Structure Staining

Leaves were first washed twice with water Type I, and then blotted dry with filter paper. Leaf pieces (2 cm^2^) from the middle of the leaf blade were embedded in 5% agar and transversal leaf cross-sections (70 μm-thick) were obtained using a vibrating blade microtome (VT1000 S, Leica Microsystems GmbH, Wetzlar, Germany). Leaf sections were stained either with safranin only (for lignin) or first with safranin and then with Alcian blue (for pectins). For safranin staining, fresh cross-sections were incubated with 0.01% (w/v) safranin for 1 min and washed three times with water Type I. After washing, some safranin-stained sections were also incubated for approximately 30 s with 50% diluted Alcian blue (1 g Alcian blue and 3 mL of glacial acetic acid in 10 mL water), and then washed three times with water Type I. Bright light images (2592 × 1994 pixels) were acquired with an inverted microscope (DM IL LED, Leica) fitted with a charge-coupled device (CCD) camera (Leica DFC 240C).

### Perls Iron Staining of Transversal Leaf Sections

The limits of detection of the Perls blue staining technique were assessed for the different compounds used as foliar fertilizers using porcelain spot plates. Tests included the compounds [FeSO_4_, Fe_2_(SO_4_)_3_, FeCl_3_, and Fe(III)-EDTA] in concentrations ranging from 5 μM to 9 mM Fe. The complexes of Fe with nicotianamine (NA), Fe(III)-NA, and Fe(II)-NA, as well as Fe(III)-citrate, were also assayed at the same concentrations. All samples (200 μL) were placed in plate wells, 20 μL of Perls dye (in 2% HCL) was added and mixed, and the blue color was developed for 20 min.

For leaf section Perls staining, leaves were excised, washed twice with water Type I and blotted dry with filter paper. Then, leaf pieces (2 cm^2^) from the middle of the leaf were embedded in 5% agar (Sigma–Aldrich, St Louis, Mo, USA) and transversal sections (70 μm-thick) were obtained using a vibrating blade microtome (VT1000 S, Leica, Germany). Fresh sections were incubated with a 2% K_4_[Fe(CN)_6_], 2% HCl solution for 30 min at room temperature. Negative stain controls were run by incubating fresh sections with HCl. Finally, sections were washed three times with water Type I reagent grade and bright light images (2592 × 1994 pixels) were taken as indicated above. Chlorophyll fluorescence images were taken with the same microscope, using an “A” Leica filter cube (excitation and emission filters BP 340–380 and LP 425, respectively).

## Results

Iron deficiency symptoms (leaf chlorosis) appeared progressively in young leaves after imposing the Fe-deficiency conditions. After 2 weeks of growth with zero Fe, plants showed marked chlorosis symptoms (**Figure 1**). Then, foliar fertilization was carried out in young, expanded Fe-deficient leaves showing chlorosis symptoms (middle leaves in **Figure 1**).

### Effects of Fe Foliar Fertilization on Leaf Fe Concentration

In all cases, treated leaves were washed thoroughly with water Type I before analysis to remove Fe remaining on the leaf surface, so that the mineral analyses reflect constitutive Fe plus any Fe from the foliar fertilizer incorporated into the leaf (e.g., incorporated in cells and/or in other leaf structures). The total Fe concentration in the leaves of Fe-deficient plants was approximately 17 μg g^-1^ DW at the beginning of the experiment (**Table 1**). All Fe foliar fertilization treatments increased markedly the leaf Fe concentrations, with the only exception of those plants treated with ABA, where the Fe concentrations did not change from the initial values (**Table 1**).

**Table 1:** Iron concentration in leaves of *Prunus dulcis × P. persica* foliar fertilized with different Fe compounds.

Time after application		Leaf Fe concentration (μg g DW^-1^)				

	**FeSO_4_**	**FeSO_4_ light-adapted**	**Fe_2_(SO_4_)_3_**	**FeCl_3_**	**Fe(III)-EDTA**	**FeSO_4_+ABA**
30 min	39.4 ± 6.2 bc^a^	49.2 ± 2.2 de	35.1 ± 0.5 b	53.5 ± 3.3 efg	55.5 ± 5.2 efg	16.2 ± 1.5 a
60 min	109.9 ± 13.7 k	112.2 ± 6.8 kl	43.2 ± 6.4 cd	58.3 ± 1.4 g	58.2 ± 3.9 g	17.5 ± 1.6 a
90 min	136.5 ± 2.5 m	136.3 ± 2.2 m	57.7 ± 4.5 fg	73.0 ± 7.3 h	54.4 ± 7.2 efg	16.4 ± 0.4 a
1 day	118.1 ± 4.11	97.2 ± 7.7 j	100.9 ± 7.9 j	82.1 ± 2.9 i	51.0 ± 6.1 ef	n.d.^b^
Treatment^c^		^∗∗∗^				
Application time		^∗∗∗^				
Treatment x Application time		^∗∗∗^				


*^a^means not sharing letters in the Table are significantly different at *p* < 0.05 (Fisher’s LSD test). ^b^n.d., not determined; ^c^ANOVA: ^∗∗∗^*p* < 0.001. Data are means ± SE (*n* = 3 plants per treatment; each sample pooled five treated leaves in each plant). All plants were illuminated for a maximum of 30 min before the treatment, except those in column 2, which were light-adapted for 2 h before the treatment. The initial Fe concentration was 16.8 ± 1.5 μg g DW^-*1*^ (*n* = 3).*

Thirty min after Fe foliar application, the leaf Fe concentration had increased two to threefold when compared to the untreated Fe-deficient leaves (**Table 1**). Iron concentrations at this time were in the range from 35 to 56 μg g^-1^ DW, with Fe(III)-EDTA and FeCl_3_ leading to the highest values and FeSO_4_ and Fe_2_(SO_4_)_3_ to the lowest ones. In the case of FeSO_4_, Fe concentrations were significantly higher when the dark pre-treatment was absent. Thirty min later (60 min after fertilizer application), leaf Fe concentrations had increased more than twofold in the case of FeSO_4_ (up to 112 μg Fe g^-1^ DW), whereas increases were minor in the case of Fe(III) salts and the Fe concentration did not change in the case of Fe(III)-EDTA. After 30 min more (90 min after fertilizer application), additional increases in leaf Fe concentrations occurred for most of the Fe compounds, with the exception of leaves treated with Fe(III)-EDTA, where the Fe concentration did not change. At this time, FeSO_4_ had led to the largest values (136–137 μg Fe g^-1^ DW), whereas Fe(III) salts led to values lower than 75 μg Fe g^-1^ DW (**Table 1**). One day after Fe application, the leaf Fe concentration had decreased somewhat for FeSO_4_, with the decrease being larger for plants without a dark pre-treatment, whereas Fe(III) salts led to further increases in leaf Fe concentrations, with the increase being larger in the case of the sulfate than the chloride form, whereas no change was found for Fe(III)-EDTA (**Table 1**).

### Localization of Xylem and Phloem Tissues in Leaf Cross-sections

**FIGURE 2:**
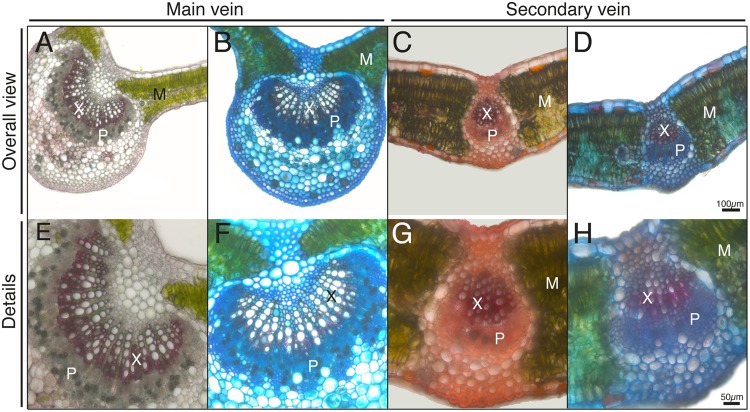
**Histological staining from transversal leaf sections.** Sections were incubated with safranin **(A,C,E,G)** or safranin + alcian blue **(B,D,F,H)**. Lignin structures are stained in red and cell walls in blue. Phloem and xylem tissues (P and X, respectively) and mesophyll tissue (M) are marked in the Figure. The scale bars correspond to 100 μm in **(A–D)** and 50 μm in **(E–H)**.

In order to localize zones corresponding to the xylem and phloem, which are rich in lignins and pectins, respectively, we used safranin and Alcian blue stains in fresh leaf sections (**Figure 2**). Safranin provides a red color and labels structures that are rich in lignins, whereas Alcian blue stains structures rich in pectins. Both in the case of the main veins (**Figures 2A,B,E,F**) and secondary ones (**Figures 2C,D,G,H**), an inner zone markedly stained with safranin was assigned to xylem tissue (marked with X in all Figures), whereas a zone surrounding the abaxial side of the xylem markedly stained with Alcian blue was assigned to phloem tissue (marked with P in all Figures). Mesophyll (M; in green due to the chlorophyll) tissue areas are also marked in the Figures.

### Assessment of Perls Staining Sensitivity

**FIGURE 3:**
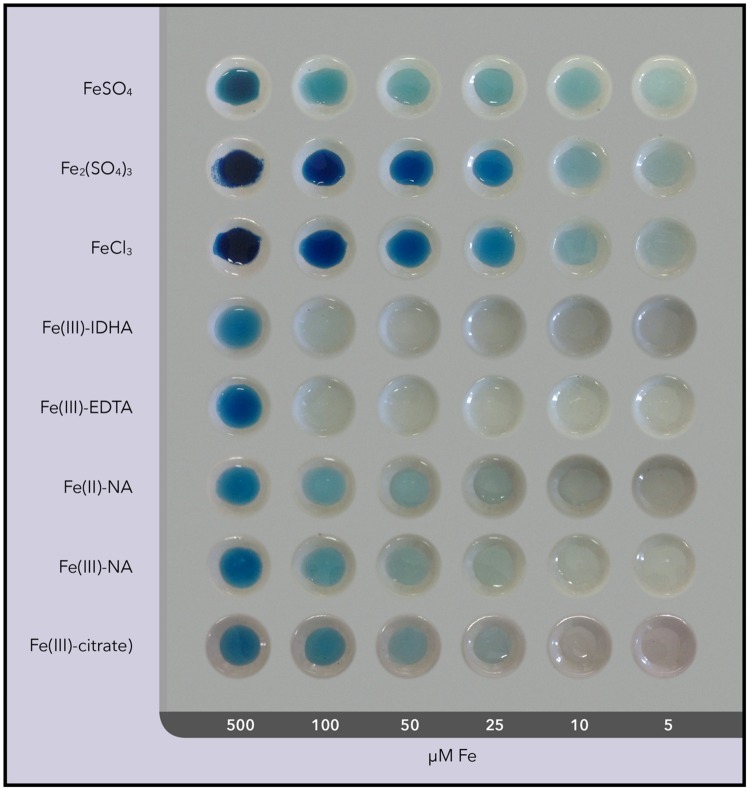
**Image of Perls blue stain with the different Fe compounds applied as foliar fertilizers and natural Fe compounds**.

The Perls Prussian blue has been reported to detect in plant tissues 35 μM Fe, irrespective of the Fe oxidation state (Roschzttardtz et al., 2009). The limits of detection for the Perls blue staining were different for the different Fe compounds used. In the case of the three Fe inorganic salts used [FeSO_4_, Fe_2_(SO_4_)_3_, and FeCl_3_] the blue stain was visible down to Fe concentrations of 5 μM (**Figure 3**). This blue precipitate is due to the formation of insoluble Iron(II,III) hexacyanoferrate(II,III). However, in the case of Fe(III)-EDTA and Fe(III)-IDHA the blue stain was scarcely visible at a Fe concentration of 100 μM. This may be ascribed to the precipitation of only a small fraction of the total Fe as Iron(II,III) hexacyanoferrate(II,III), with most of the Fe being still chelated by the chelating agent. To assess the possible detection of natural Fe chelates, Fe-NA complexes and Fe-citrate were also assayed, and the limits of detection for the Perls blue staining were lower than 25 μM Fe for Fe(II)-NA, Fe(III)-NA, and Fe-citrate (**Figure 3**).

### Perls Staining of Tissue Sections

**FIGURE 4:**
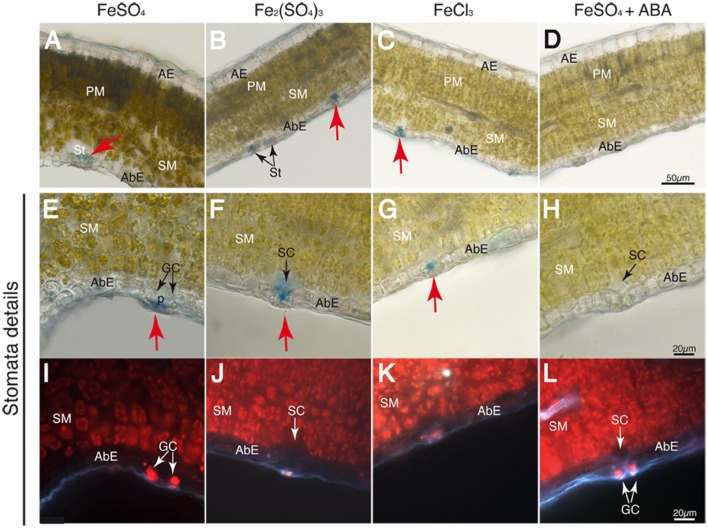
**Localization of labile Fe pools using Perls blue staining in transversal leaf sections taken 30 min after Fe foliar fertilization: leaf blades **(A–D)**, close-up of stomatal areas **(E–H)** and chlorophyll fluorescence image of the same fields in **E–H**, revealing the location of stomatal guard cells and mesophyll cells **(I–L)** Leaves were treated with FeSO_4_**(A,E,I)**, Fe_2_(SO_4_)_3_**(B,F,J)**, FeCl_3_**(C,G,K)**, and FeSO_4_ in ABA-treated plants **(D,H,L)**.** Abaxial and adaxial epidermis (AbE and AE), parenchima and spongy mesophyll tissue (PM and SM), stomata (St) and stomatal guard cells (GC), cavities (SC), and pores (p) are marked in the images. The scale bars correspond to 50 and 20 μm in **(A–D)** and **(E–L)**, respectively.

**FIGURE 5:**
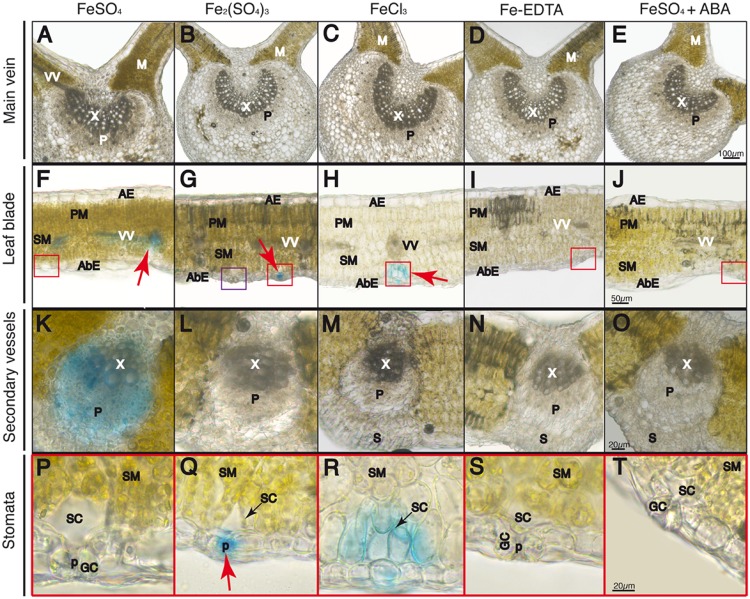
**Localization of labile Fe pools using Perls blue staining in transversal leaf sections taken 60 min after Fe foliar fertilization: main vein **(A–E)**, leaf blades **(F–J)**, close-up of minor veins in **F–J** (**K–O**) and close-up of stomatal areas in red squares in **F–J(P–T)**.** Leaves were treated with FeSO_4_ **(A,F,K,P)**, Fe_2_(SO_4_)_3_ **(B,G,L,Q)**, FeCl_3_ **(C,H,M,R)**, Fe(III)-EDTA **(D,I,N,S)**, and FeSO_4_ in ABA-treated plants **(E,J,O,T)**. Phloem and xylem tissues (P and X, respectively) and mesophyll tissue (M) are marked in images **(A–E)** and **(K–O)**; abaxial and adaxial epidermis (AbE and AE), parenchima and spongy mesophyll tissue (PM and SM), and stomatal guard cells (GC), cavities (SC), and pores (p) and vascular vessels (VV) are marked in images **(F–J)** and **(P–T)**. The scale bars correspond to 100, 50, and 20 μm in **(A–E)**, **(F–J)**, and **(K–T)**, respectively.

**FIGURE 6:**
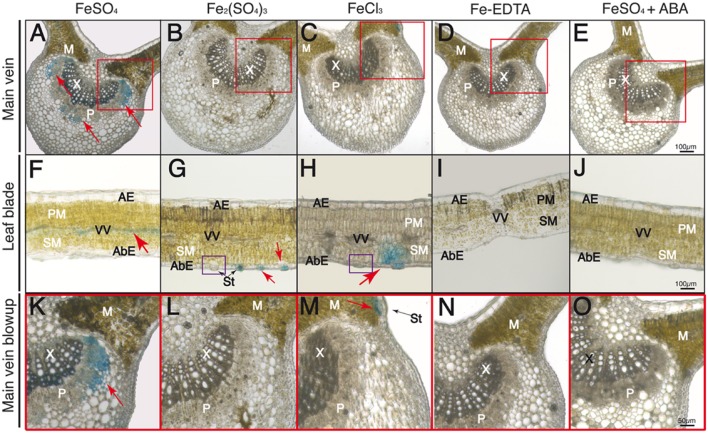
**Localization of labile Fe pools using Perls blue staining in transversal leaf sections taken 90 min after Fe foliar fertilization: main vein **(A–E)**, leaf blades **(F–J)** and close-up of main vein areas in red squares in **(A–E)(K–O)**.** Leaves were treated with FeSO_4_ **(A,F,K)**, Fe_2_(SO_4_)_3_ **(B,G,L)**, FeCl_3_ **(C,H,M)**, Fe(III)-EDTA **(D,I,N)** and FeSO_4_ in ABA-treated plants **(E,J,O)**. Phloem and xylem tissues (P and X, respectively) and mesophyll tissue (M) are marked in images **(A–E)** and **(K–O)**; abaxial and adaxial epidermis (AbE and AE), parenchima and spongy mesophyll tissue (PM and SM), stomata (St) and vascular vessels (VV) are marked in images **(F–J)**. Purple squares in **(G,H)** indicate stomata without blue stain. The scale bars correspond to 100 and 50 μm in **(A–J)** and **(K–O)**, respectively.

**FIGURE 7:**
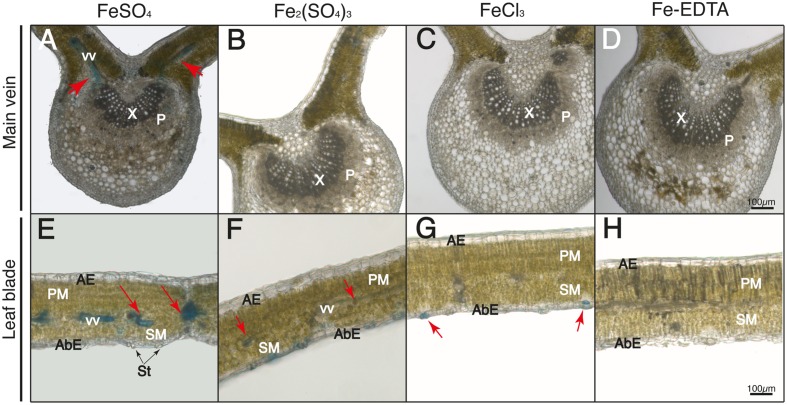
**Localization of labile Fe pools using Perls blue staining in transversal leaf sections taken 24 h after Fe foliar fertilization: main vein **(A–D)** and leaf blades **(E–H)** Leaves were treated with FeSO_4_**(A,E)**, Fe_2_(SO_4_)_3_**(B,F)**, FeCl_3_**(C,G)**, and Fe(III)-EDTA **(D,H)**.** Phloem and xylem tissues (P and X, respectively) and vascular vessels (VV) are marked in images **(A–D)**; abaxial and adaxial epidermis (AbE and AE), parenchima and spongy mesophyll tissue (PM and SM), stomata (St) and vascular vessels (VV) are marked in images **(E–H)**. Scale bars correspond to 100 μm.

Leaf cross-sections were observed after Perls staining by optical microscopy. The adaxial and abaxial epidermis (AE and AbE, respectively), pallisade and spongy mesophyll (PM and SM, respectively), stomata (St), and the stomatal cavity and guard cells (SC and GC, respectively) are labeled in **Figures 4–7**.

The Perls blue precipitate Fe staining was observed in some cases but only in two zones, the stomatal and vascular areas, and this was dependent on the Fe compound used and also on the time after application. A blue color was observed in stomatal areas with some treatments but only in plants that were dark-adapted (i.e., covered with a black plastic bag overnight), and then illuminated for a maximum of 30 min before foliar application. When plants were exposed to the growth chamber light for a longer period of time (i.e., 2 h or more), a Perls blue stain was observed in the vascular areas in some cases, but never in the stomatal areas with any of the treatments used. Unless otherwise stated, the observations described below refer to plants covered with a black plastic bag overnight and illuminated for only 30 min.

At 30 min after fertilizer application, the Perls blue color appeared only in the stomata (red arrows), whereas no stain was observed in the mesophyll or vascular vessels with any of the Fe forms applied (**Figures 4A–C**). An exception was the FeSO_4_+ABA treatment, where no Perls blue color was observed anywhere (**Figure 4D**). When using a higher magnification, stomata appeared open and the blue Fe color was observed in the stomatal pore and/or the sub-stomatal cavity in all treatments (**Figures 4E–G**), with the exception of the plants pre-treated with ABA (**Figure 4H**). With regard to Fe(III)-EDTA, we did not observe any blue stain (data not shown). Chlorophyll fluorescence images of the same tissue preparations shown in **Figures 4E–H** reveal -in red- the location of the stomatal guard cells (GC) as well as the inner spongy mesophyll cells (SM) (**Figures 4I–L**). When plants were illuminated for 2 h or more prior to Fe fertilization, some stain appeared in the vascular areas (not shown), but the stain was never found in the stomatal areas.

At 60 min after fertilizer application, no Perls blue staining was observed in the main leaf vein (**Figures 5A–E**). However, in the case of the FeSO_4_ treatment some blue staining appeared in the minor leaf veins and the vascular bundles (red arrow in **Figure 5F**), whereas the stain was no longer present in the stomatal area (red square area in **Figure 5F**, see blow-up in **Figure 5P**). Observing the minor veins with a higher magnification, it appears that the blue color is detected both in the phloem and xylem tissue, with the highest intensity being found in part of the phloem vessels (**Figure 5K**). In the case of Fe_2_(SO_4_)_3_, we did not observe any changes from the image obtained at 30 min after Fe application, since the Perls blue stain still remained in the stomatal area (red square area in **Figure 5G**, see blow-up in **Figure 5Q**). A non-stained stomata is also seen in the purple square in **Figure 5G**. When Fe was applied as FeCl_3_, the blue color distribution was somewhat similar to that obtained with the other Fe(III) salt used, Fe_2_(SO_4_)_3_ (**Figures 5C,H,M,R**), although the stain had moved slightly from the sub-stomatal cavity to neighboring spongy mesophyll cells (**Figure 5R**). With regard to Fe(III)-EDTA, we did not observe any blue stain (**Figures 5D,I,N,S**). Finally, when the plants were pre-treated with ABA before FeSO_4_ application we did not observe blue staining anywhere and stomata appeared closed (**Figures 5E,J,O,T**). When plants illuminated for 2 h or more were used with FeSO_4_, the blue stain was only found in vascular tissues (not shown).

At 90 min after fertilizer application, Perls blue staining was observed in the main vein in the case of FeSO_4_ (**Figure 6A**), with additional blue stain being found along the vascular tissues (**Figure 6F**). Using a higher magnification the stain appeared localized in the phloem tissue and also in some xylem areas (red square area in **Figure 6A**, see blow-up in **Figure 6K**). No stain was observed in the main vein with the other Fe compounds (**Figures 7B–D**). On the other hand, in the case of the Fe(III) salts the Perls blue stain did not change when compared to the 60 min image (**Figures 6G,H**). Non-stained stomata are also seen in the purple squares in **Figures 6G,H**. No blue stain was observed in plants treated with Fe(III)-EDTA (**Figures 6D,I,N**) or pre-treated with ABA before FeSO_4_ application (**Figures 6E,J,O**).

After 24 h, in the case of FeSO_4_ the Perls blue staining was localized in vascular tissues (red arrows in **Figures 7A,E**), with less staining in the central vein when compared to the 90 min images. In the case of Fe_2_(SO_4_)_3_ the stain was present not only in stomatal areas but also in some areas of the vascular bundles (red arrows in **Figure 7F**), with no blue stain being observed inside the central vein (**Figure 7B**). In the case of FeCl_3_ the Perls blue stain was only present in the stomatal area (**Figures 7C,G**). Again, no blue stain was observed for Fe(III)-EDTA (**Figures 7D,H**).

## Discussion

Results show that the Perls blue staining method is useful for tracing the Fe uptake pathway in leaves, since it is capable to detect, using 70-μm leaf tissue sections, new labile Fe pools originated in the stomatal and vascular leaf areas after foliar fertilization. *In vitro* experiments indicate that the limit of detection can be as low as 5 μM with Fe inorganic salts, 10–25 μM with Fe-complexes with natural, endogenous chelating agents such as NA and citrate and 100 μM with synthetic Fe-chelates such as Fe(III)-EDTA and Fe(III)-IDHA. The detection of Fe in stomatal and vascular areas in leaves treated with Fe inorganic salts (both Fe(II) and Fe(III)) supports that the new Fe labile pools occurring as a result of foliar Fe application of these compounds are in concentrations >10 μM. However, since the Perls stain cannot provide information on Fe speciation, the detected new Fe pools may include either the original Fe species applied or in any of the natural Fe(II) or Fe(III) complexes that can be synthesized within the plant [e.g., Fe(II)-NA, Fe(III)-NA or Fe-citrate], given that all of them can be also detected with the Perls method at concentrations lower than 25 μM. In the case of fertilization with Fe(III)-EDTA, the lack of Perls stain supports that Fe concentrations >100 μM were not reached in any leaf area. The fact that Perls blue stain was never detected in the leaf mesophyll areas in the Fe-treated leaves is likely because labile Fe concentrations in this area are lower than 10 μM (see below).

Results show that Fe applied as inorganic salts was taken up through the stomata, and that this occurred rapidly, within 30 min of the application. By this time leaf Fe concentrations had increased two to threefold (depending on the fertilizer) with Fe(II) in the form of sulfate, as well as with Fe(III) in the forms of sulfate and chloride. Conversely, in the case of Fe(III)-EDTA an increase in leaf Fe occurred but no visible stains are evident (images not shown), as it could be expected from the poor limit of detection for this compound with the Perls method. The role of stomata in Fe uptake was confirmed using ABA, since ABA-induced stomatal closure fully inhibited Fe uptake, as shown both by the unchanged leaf Fe concentration and the absence of Perl blue stains in the images. These data also support that entrance though the cuticle did not occur, at least in plants pre-treated with ABA. These results confirm the previous observation that Fe uptake occurs *via* stomata (e.g., Middleton and Sanderson, 1965; Eichert et al., 1998, 2002; Schlegel and Schönherr, 2002; Fernández et al., 2005; Schlegel et al., 2005, 2006), and is in line with the suggestion that stomata could provide a pathway for Fe uptake from the leaf surface to the sub-stomatal cavity (Eichert and Burkhardt, 2001). Since the resolution of the images when using 70 μm-thick sections is limited, we cannot ascertain precisely whether the applied Fe entered leaves through mass flow or diffusion via wall surfaces.

There were major differences in Fe uptake among the inorganic Fe compounds tested, with FeSO_4_ being the most effective. The foliar application of FeSO_4_ led to a progressive appearance of the Perls blue stain, first in stomatal areas (at 30 min), then in leaf blade vascular tissues (at 60 min) and finally in the central vascular tissues (at 90 min), with the initial Perls blue stain in the stomatal areas disappearing gradually. After 1 day, the stain in the central leaf vein had also faded, although the vascular tissues in the leaf vein were still stained. This pattern suggests the occurrence of labile (Perls-reacting) Fe pools in the stomatal areas that can be fully re-mobilized in less than 2 h. This sequence of events was accompanied by twofold and 13% increases (at 60 and 90 min) and a 30% decrease (at 24 h) in leaf blade Fe concentration. After 24 h, some Perls blue stain was still present in vascular tissues in the leaf blade, indicating the occurrence of some labile but non-mobile Fe pools in the vascular tissue. These results are in line with previous studies indicating that FeSO_4_ is a good foliar fertilizer (Rombolà et al., 2000; Pestana et al., 2001, 2003; Álvarez-Fernández et al., 2004; Fernández et al., 2006, 2008; Borowski, 2013). However, in the case of Fe(III) salts the Perls blue color was mostly restricted to the stomatal areas, although after 24 h some blue traces were also present in the vascular areas. The leaf Fe concentration increase with the two Fe(III) salts used was much lower than those with FeSO_4_ in the short term, but afterward it increased progressively with time. This pattern suggests that the free Fe pools formed in stomata upon fertilization with Fe(III) salts were less mobile than those formed upon fertilization with Fe(II).

The differences found among the products tested could be potentially ascribed to the effects of ionic charge, molecular size and pH on the formation of Fe oxyhydroxides as Perls-reactive labile leaf Fe pools. Regarding charge, major ionic species in equilibrium at moderately acidic pH will be the +2 ion in the case of Fe(II) and +1 hydroxylated species in the case of Fe(III), respectively (Lindsay, 1995), whereas the -1 charged species will be prevalent in the case of Fe(III)-EDTA (Lindsay, 1979). Regarding molecular size, the mean maximum molecular radii of the Fe species likely to occur in the Fe formulations used are approximately 0.1 nm for Fe^2+^ and <1 nm for Fe^3+^ hydroxylated species and Fe(III)-EDTA (Fernández et al., 2008), sizes much smaller than the aperture of the stomata and below limits described for the transport of substances in the cuticle of a few species (ranging between 0.5 and 2.4 nm; Schönherr, 2006; Eichert and Goldbach, 2008). Regarding pH, a progressive appearance of oxyhydroxides would be expected, since Fe taken up must travel through a pH gradient, from the acidic fertilizer solutions (pH 3.9, 3.2–3.3, and 4.1 in the cases of FeSO_4_, Fe(III)salts and Fe(III)EDTA, respectively) toward the moderately acidic pH values generally prevalent in the leaf sub-stomatal chamber (5.0; Felle and Hanstein, 2002), and the less acidic values in the apoplastic fluid (5.5–6.5 in sugar beet, López-Millán et al., 2000; Larbi et al., 2010) and xylem sap (6.5–7.0 in peach, Larbi et al., 2003; 5.7–6.2 in sugar beet, López-Millán et al., 2000; Larbi et al., 2010).

Also, the presence in the different compartment of natural chelating agents such as NA and citrate as well as cell wall and plasma membrane interactions are likely to be relevant. In the case of the complexes with NA, the predominant ionic species will be the neutral molecule in the case of Fe(II)-NA and both the neutral molecule and +1 ion species in the case of Fe(III)-NA (Rellán-Álvarez et al., 2010). In the cases of the complexes with citrate, the predominant ionic species will be +2 ion species in the case of the Fe_3_-Citrate_3_ and +2 and +1 species in the case of Fe_2_-Citrate_2_ (Rellán-Álvarez et al., 2010). Concentrations reported so far are generally lower for NA [5–20 μM in tomato apoplastic fluid (Díaz-Benito, Unpublished Data) and 0–271 μM in the xylem sap of different species (Álvarez-Fernández et al., 2014)] than for citrate [0.2–1.8 mM in apoplastic fluid of different species (Álvarez-Fernández et al., 2014) and 0.1–0.8 mM in peach xylem sap (Larbi et al., 2003)]. A tentative explanation for the permanent Perls stain in stomata of leaves treated with Fe(III) can be inferred from *in vitro* citrate/NA ligand competition studies, which show that in the presence of citrate and at an acidic pH value (5.5) Fe(II)-NA can still occur whereas Fe(III)-NA would be absent due to the preferential formation of Fe-citrate complexes (Rellán-Álvarez et al., 2008). A possible hypothesis would be that Fe in the substomatal cavity can be transported more efficiently when Fe(II) is used because of the action of a so far uncharacterized Fe(II)-NA transporter. However, the complete lack of data for the citrate and NA concentrations in the substomatal cavity and the complexity of the Fe chemistry in aerobic environments (Pierre and Gautier-Luneau, 2000; Fernández and Ebert, 2005; Gautier-Luneau et al., 2005) do not allow to fully explain data found in this study.

Results show that in the conditions used in the experiment (70% RH) Fe was taken up more efficiently when applied as Fe salts than when applied as Fe(III)-EDTA. This is likely due to the lower POD of Fe salts (<60%; Schönherr, 2002) when compared to those of Fe(III)-EDTA (near 100% RH) (Schönherr et al., 2005). At humidity values of 70% or lower (values similar to those commonly found in field conditions in areas affected by Fe chlorosis) the uptake of Fe from any compound with high POD values will be limited only to the periods when they are present in liquid form (i.e., immediately after application and before fertilizer drying), whereas in the case of Fe salts the lower POD will allow for a more extended uptake period. Although it has been proposed that once in the apoplast non-charged Fe forms could be translocated in the apoplast more easily than positively charged ones (Fernández et al., 2005), our data show that Fe ionic forms are easily taken up and translocated, perhaps after conversion into neutral forms. Therefore, the view that foliar-applied chelates such as Fe(III)-EDTA could be translocated more readily than ionic Fe-containing substances (Hsu and Ashmead, 1984; Fernández et al., 2005) should be taken with caution.

Results showing a rapid intra-leaf mobility of Fe in foliar fertilized leaves are in contrast with the widely accepted view that foliar Fe fertilization has only local effects in the foliage directly treated (Zhang and Brown, 1999; Fernández et al., 2013; El-Jendoubi et al., 2014) due to the poor Fe mobility into other plant organs (White, 2012). These apparently contrasting results can be reconciled by two reasons. First, intra-leaf mobility can be overestimated with the regular non-enhanced Perls method, because the Fe-deficient mesophyll tissue can take up avidly Fe upon fertilization, leading to free Fe concentrations in the bulk mesophyll below the 5 μM detection range. This is confirmed by the fact that when the more sensitive Perls diaminobenzidine-enhanced method (Perls-DAB) was used in FeSO_4_-treated leaves the whole leaf mesophyll tissue was stained (El-Jendoubi et al., 2014). Second, there is new evidence for Fe long distance mobility after foliar fertilization, although this may be species-dependent. Recent studies have shown that some of the Fe taken up by leaves, 1–12% depending on the fertilizer and the plant species, can move from the treated leaves toward the roots (Nikolic et al., 2003; Rodríguez-Lucena et al., 2009, 2010; Roosta and Mohsenian, 2012; Zhang et al., 2013; Carrasco-Gil et al., 2016). In tomato, it has been proposed that this occurs *via* phloem transport, since phloem tissues are stained by Perls blue shortly after foliar fertilization (Carrasco-Gil et al., 2016).

An interesting issue is why no Perls blue stain is visible in the stomatal areas when FeSO_4_ was applied to plants illuminated for 2 h or more. A likely explanation is that in Fe-deficient plants light-adapted for 2 h or more the uptake and/or transport processes could be primed and thereby be faster than in leaves coming from a prolonged dark period. This priming would lead to a faster Fe uptake into cells and organelles and transport, so that no labile (Perls-detectable) Fe pools would occur in the stomatal areas. The existence of this priming effect is supported by the fact that increases in leaf Fe concentration 30 min after foliar treatment were larger in leaves light-adapted for 2 h than in those coming from dark adaptation (31 vs. 20 μg Fe g^-1^ DW). Furthermore, at 60 and 90 min, the leaf Fe concentrations became similar in both plant types, whereas after 1 day the leaf Fe concentration was lower in plants treated after light-adaptation than in those treated after dark-adaptation.

In summary, we have shown that the Perls blue method is a good tool to trace the Fe uptake pathway in leaves when using Fe salts, but is not sensitive enough when using synthetic Fe(III)-chelates such as Fe(III)-EDTA or Fe(III)-IDHA. Foliar Fe fertilization increased leaf Fe concentrations with all Fe compounds used, with inorganic Fe salts being more efficient than Fe(III)-EDTA. Results show that Fe applied as inorganic salts was taken up rapidly through the stomata. In the case of using FeSO_4_ a progression of the stain was seen with time toward vascular areas in the leaf blade and the central vein, whereas in the case of Fe(III) salts the stain remained in the stomatal areas. The Perls method is cheap and accessible in many research and industrial laboratories and does not require expensive, dedicated instrumentation as it occurs for other metal image analysis techniques. Results in this study open the possibility to easily test new Fe fertilizer formulations, as well as to study the possible Fe transporters responsible for leaf Fe uptake.

## Author Contributions

JR and SC-G carried out experiments, JR, SC-G, AA, and JA planned research, JR and JA wrote the paper.

## Conflict of Interest Statement

The authors declare that the research was conducted in the absence of any commercial or financial relationships that could be construed as a potential conflict of interest.
